# Composite Polymer Electrolytes with Tailored Ion-Conductive Networks for High-Performance Sodium-Ion Batteries

**DOI:** 10.3390/ma18133106

**Published:** 2025-07-01

**Authors:** Caizhen Yang, Zongyou Li, Qiyao Yu, Jianguo Zhang

**Affiliations:** School of Mechatronic Engineering, Beijing Institute of Technology, Beijing 100081, China; 3120240251@bit.edu.cn (C.Y.); 3220215031@bit.edu.cn (Z.L.)

**Keywords:** gel-polymer electrolyte, sodium-ion batteries, perovskite, polymer–inorganic composite

## Abstract

Gel-polymer electrolytes offer a promising route toward safer and more stable sodium-ion batteries, but conventional polymer systems often suffer from low ionic conductivity and limited voltage stability. In this study, we developed composite GPEs by embedding methylammonium lead chloride (CH_3_NH_3_PbCl_3_, MPCl) into a UV-crosslinked ethoxylated trimethylolpropane triacrylate (ETPTA) matrix, with sodium alginate (SA) as an ionic conduction enhancer. Three types of membranes—GPE-P, GPE-El, and GPE-Eh—were synthesized and systematically compared. Among them, the high-MPCl formulation (GPE-Eh) exhibited the best performance, achieving a high ionic conductivity of 2.14 × 10^−3^ S·cm^−1^, a sodium-ion transference number of 0.66, and a wide electrochemical window of approximately 4.9 V vs. Na^+^/Na. In symmetric Na|GPE|Na cells, GPE-Eh enabled stable sodium plating/stripping for over 600 h with low polarization. In Na|GPE|NVP cells, it delivered a high capacity retention of ~79% after 500 cycles and recovered ~89% of its initial capacity after high-rate cycling. These findings demonstrate that the perovskite–polymer composite structure significantly improves ion transport, interfacial stability, and electrochemical durability, offering a viable path for the development of next-generation quasi-solid-state sodium-ion batteries.

## 1. Introduction

With the rapid expansion of renewable energy and the increasing demand for efficient energy storage systems, there is an urgent need to develop low-cost, safe, and scalable battery technologies. Sodium-ion batteries (SIBs) have attracted significant attention as a promising alternative to lithium-ion batteries (LIBs), due to the natural abundance, wide distribution, and low cost of sodium resources. These features make SIBs especially suitable for large-scale energy storage, low-temperature applications, and off-grid power supply in remote areas [1,2,3,4,5].

Although considerable progress has been made in developing suitable electrode materials for SIBs, electrolyte systems remain a bottleneck for their commercialization. Conventional liquid electrolytes, while offering high ionic conductivity, are volatile, flammable, and thermally unstable. These characteristics pose serious safety risks during long-term cycling, high-temperature operation, or accidental leakage. Gel-polymer electrolytes, on the other hand, have garnered significant interest owing to their non-flammability, mechanical robustness, flexibility, and potential to suppress dendritic growth, offering a viable pathway to safer and more reliable SIB systems [3,6,7,8].

Among various polymer matrices studied, polyethylene oxide (PEO) is widely used due to its excellent processability and sodium salt solubility. However, the high crystallinity of PEO at ambient temperature severely limits its ionic mobility and results in low ionic conductivity (~10^−5^–10^−6^ S/cm), poor interfacial contact, and inadequate long-term cycling stability [9,10,11,12,13]. To address this, researchers have explored amorphous or low-crystallinity polymer alternatives to facilitate ion transport.

Ethoxylated trimethylolpropane triacrylate (ETPTA), a trifunctional acrylate monomer, has emerged as a promising candidate. Under ultraviolet (UV) irradiation, ETPTA rapidly crosslinks into a dense, highly amorphous three-dimensional network. This crosslinked structure not only suppresses crystallization but also improves mechanical stability and provides continuous ion-conductive pathways [14,15,16,17]. Recent studies have demonstrated that ETPTA-based electrolytes can achieve ionic conductivities as high as 10^−4^ S/cm at room temperature, significantly outperforming PEO-based systems [8,18,19,20].

In addition to optimizing the polymer backbone, the incorporation of functional inorganic additives has proven effective in enhancing GPE performance. Methylammonium lead chloride (CH_3_NH_3_PbCl_3_, abbreviated as MPCl), a perovskite-structured compound originally studied in photovoltaics, possesses a highly ordered crystal lattice, low ion migration barriers, and intrinsic ionic conductivity [21,22,23,24]. When introduced into polymer matrices, MPCl can serve as a fast ion conductor by constructing efficient percolation pathways and enhancing sodium ion mobility [25,26,27]. Furthermore, its presence may help stabilize polymer/electrode interfaces through electrochemical insulation and spatial buffering effects [28,29,30].

To further improve mechanical properties and film-forming ability, sodium alginate (SA), a natural biopolymer with abundant carboxyl functional groups, is incorporated into the polymer matrix. SA contributes to the structural integrity of the composite membrane while enhancing ion coordination and transport pathways due to its metal ion chelation capacity [31,32].

In this study, we designed and synthesized three different gel-polymer electrolyte systems: (1) PEO + MPCl + SA; (2) UV-cured ETPTA + MPCl + SA; and (3) UV-cured ETPTA + high-concentration MPCl + SA. By systematically comparing their thermal stability, ionic conductivity, electrochemical stability, and interfacial compatibility, we aim to elucidate the synergistic effects of the polymer network structure and perovskite-type filler concentration on the overall electrolyte performance. This work provides new design insights and experimental evidence for the development of high-performance gel-polymer electrolytes for next-generation sodium-ion batteries.

## 2. Materials and Methods

### 2.1. Materials and Reagents

All reagents used in this study were used without further purification. Polyethylene oxide (PEO, Mw ≈ 600,000), sodium alginate (SA), and lead(II) chloride (PbCl_2_) were purchased from Zancheng (Tianjin) Technology Co., Ltd. (Tianjin, China). N,N-dimethylformamide (DMF) and methanol (MeOH) were obtained from Aladdin Reagents. (Shanghai, China). Ethoxylated trimethylolpropane triacrylate (ETPTA) and 2-hydroxy-2-methylpropiophenone (HMPP, the photoinitiator) were supplied by Shanghai Macklin Biochemical Co., Ltd., (Shanghai, China). Methylammonium chloride (MACl) was also obtained from Macklin and used to synthesize methylammonium lead chloride (CH_3_NH_3_PbCl_3_, MPCl) in the lab. Polyvinylidene fluoride (PVDF, Mw ≈ 500,000) was purchased from Shanghai D&B Biological Science and Technology Co., Ltd., (Shanghai, China). The conductive carbon black (Super C 45) was obtained from Guangdong ZhuGuang New Energy Technology Co., Ltd., (Nanxiong, China). Spherical hard carbon for sodium-ion batteries was purchased from Youyan Technology Co., Ltd., (Beijing, China). Sodium vanadium phosphate (Na_3_V_2_(PO_4_)_3_, NVP) and sodium metal were purchased from Alfa Aesar (Shanghai, China). The commercial liquid electrolyte (1 M NaClO_4_ in PC, DEC:EC = 1:1 *v*/*v*, with 5 wt% fluoroethylene carbonate (FEC), product code NC-013) was purchased from DoDoChem Co., Ltd., (Suzhou, China).

### 2.2. Preparation of Perovskite Electrolyte

The synthesis method in reference [21] was adopted and optimized for the purpose of this experiment. MACl and PbCl_2_ with a molar ratio of 1:1 were dissolved in a mixed solvent of DMF/DMSO with a volume ratio of 1:1 at a concentration of 1 M. The solution was stirred at room temperature until it became clear and transparent. Subsequently, it was stirred at 80 °C for 4 h, filtered through a 22 μm pore-sized polytetrafluoroethylene filter, washed, and then dried to obtain MAPbCl_3_ (MPCl) crystal powder, which was stored in a dry environment.

### 2.3. Preparation of Gel-Polymer Electrolyte Membranes

Three types of gel-polymer electrolyte (GPE) membranes were prepared: a PEO-based system (GPE with PEO, GPE-P), a low-MPCl-content ETPTA-based system (GPE with ETPTA and low-MPCl-content, GPE-El), and a high-MPCl-content ETPTA-based system (GPE with ETPTA and high-MPCl-content, GPE-Eh). For the PEO-based membrane, 800 mg of PEO, 350 mg of MPCl, and 100 mg of SA (weight ratio 8:3.5:1) were dispersed in deionized water under vigorous magnetic stirring at 60 °C for 4 h to obtain a homogeneous solution. The resulting mixture was cast onto a clean glass substrate and dried under vacuum at 60 °C for 12 h to form a flexible membrane.

For the ETPTA-based membranes, MPCl and SA were first dispersed in DMF, followed by the addition of ETPTA monomer and 3 wt% HMPP as the photoinitiator (relative to the total monomer mass). In the low-concentration MPCl system, the mass ratio of ETPTA:MPCl:SA was 4:3.5:1 (i.e., 400 mg ETPTA, 350 mg MPCl, 100 mg SA in 5 mL DMF). For the high-concentration MPCl system, the ratio was 4:10.5:1 (i.e., 400 mg ETPTA, 1050 mg MPCl, 100 mg SA in 10 mL DMF). More extreme formulations, such as ETPTA:MPCl:SA = 4:21:1, were also tested, but the resulting membranes exhibited severe cracking after electrolyte soaking due to excessive filler content, as shown in Figure S1. Therefore, 4:10.5:1 was selected as the optimal composition. After stirring at room temperature for 6 h, the precursor solutions were cast onto glass substrates and exposed to 365 nm UV light for 10 min to induce polymerization. The resulting films were carefully peeled off and further dried under vacuum at 60 °C to remove residual solvent.

Before use, the polymer membrane was placed in a glovebox and soaked in the liquid electrolyte (NC-013) for 12 h to obtain the usable electrolyte membrane. Finally, the membrane was cut into circular pieces with a diameter of 16 mm. The final thickness of the membranes after drying and electrolyte soaking was measured to be in the range of 170–210 μm, depending on composition and solvent content. The GPE-Eh membrane had an average thickness of approximately 200 μm. The liquid electrolyte uptake (U) of GPE-Eh was approximately 38.6 wt%. This experiment was carried out in an argon atmosphere to avoid the influence of water and air on the electrolyte.

### 2.4. Materials Characterization

The microstructure and surface morphology of the membrane samples were characterized by field emission scanning electron microscopy (SEM, JSM-7800F, JEOL Ltd., Tokyo, Japan) operated at an accelerating voltage of 5 kV. The crystalline structures of the polymer matrix and embedded MPCl phase were analyzed using X-ray diffraction (XRD, D8 ADVANCE, Bruker Corporation, Karlsruhe, Germany) with Cu-Kα radiation in the 2θ range of 5° to 70°. Chemical functionalities and polymerization features were identified via Fourier transform infrared spectroscopy (FTIR, Nicolet 6700, Thermo Fisher Scientific, Waltham, MA, USA) in the wavenumber range of 600–4000 cm^−^^1^. Thermal behavior and stability were evaluated using differential scanning calorimetry–thermogravimetric analysis (DSC-TG, Mettler Toledo, Greifensee, Switzerland) under nitrogen flow with a heating rate of 10 °C/min from 25 °C to 500 °C.

### 2.5. Electrochemical Measurements

The electrolyte uptake (U) was calculated using Equation (1):

(1) $$U=\frac{M_{2}-M_{1}}{M_{1}}\times 100\%$$

where *M*_1_ and *M*_2_ represent the sample weights before and after liquid electrolyte absorption, respectively.

The ionic conductivity (*σ*) was measured via electrochemical impedance spectroscopy (EIS) using a stainless steel | MSE | stainless steel (SS | MSE | SS) cell configuration, and calculated by Equation (2):

(2) $$\sigma =\frac{l}{RS}$$

where l is the membrane thickness, R is the bulk resistance obtained from EIS, and S is the contact area.

The activation energy (*E_a_*) was obtained by fitting temperature-dependent conductivity data using the Arrhenius equation:

(3) $$\sigma =\sigma_{0}\times e^{-\frac{E_{a}}{RT}}$$

The sodium-ion transference number (*t_N__a_^+^*) was determined using DC polarization with a Na | MSE | Na symmetric cell and calculated by the Bruce–Vincent equation:

(4) $$t_{{Na}^{+}}=\frac{I_{S}\left(\Delta V-I_{0}R_{0}\right)}{I_{0}\left(\Delta V-I_{s}R_{S}\right)}$$

where *I*_0_ and *I_s_* are the initial and steady-state currents, *R*_0_ and *R_s_* are the corresponding interfacial resistances, and Δ*V* is the applied potential (10 mV).

The electrochemical stability window was measured by linear sweep voltammetry (LSV) using the CHI660E workstation, scanned from 0 to 6 V (vs. Na^+^/Na) at 1 mV s^−1^ with stainless steel as the working electrode and sodium metal as the counter/reference electrode. Cyclic voltammetry (CV) was also performed in the range of −1 to 4 V (vs. Na^+^/Na) at a scan rate of 1 mV s^−1^ under the same configuration.

To evaluate interfacial compatibility and polarization behavior between GPEs and metallic sodium, Na | GPE | Na symmetric cells were assembled and tested for impedance evolution over storage time and for sodium plating/stripping stability. The sodium foil diameter was 14 mm with an active area of 1.54 cm^2^.

Cells were assembled in an argon-filled glovebox using sodium vanadium phosphate (NVP) as the cathode and metallic sodium as the anode. The cathode slurry was composed of NVP, conductive carbon, and PVDF in a weight ratio of 7:2:1, with an NVP mass loading of approximately 0.78 mg cm^−2^. Cell tests were performed on a LAND battery tester, and CV scans were conducted at 0.2 mV s^−1^.

## 3. Results and Discussion

**Figure 1:**
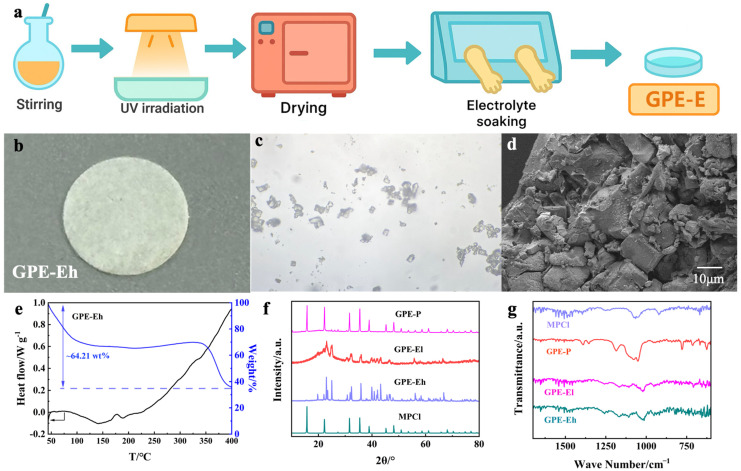
(**a**) Schematic illustration of GPE-E membrane preparation via stirring, UV curing, drying, and electrolyte soaking in a glovebox. (**b**) Photograph of prepared GPE-Eh membrane. (**c**) Optical microscope image of MPCl particles. (**d**) SEM image of the fractured cross-section of GPE-Eh. (**e**) TG–DSC curves of GPE-Eh. (**f**) XRD patterns of MPCl, GPE-P, GPE-El, and GPE-Eh. (**g**) FTIR spectra of MPCl and composite electrolyte membranes.

The preparation process of the GPE-E membrane is illustrated in Figure 1a. The precursor solution containing ETPTA, MPCl, SA, and photoinitiator was first stirred to form a homogeneous dispersion, followed by UV irradiation to induce rapid crosslinking. After polymerization, the membrane was vacuum-dried and subsequently transferred to an argon-filled glovebox for liquid electrolyte soaking using a petri dish to ensure full infiltration. The resulting membrane (Figure 1b) exhibits a uniform and flexible film, suitable for coin cell assembly.

The microstructure of MPCl crystals is shown in the optical image (Figure 1c), revealing cubic-like morphologies typical of perovskite structures. SEM observation of the cross-section of GPE-Eh (Figure 1d) indicates a dense, rough fractured surface with embedded granules, suggesting the successful integration of MPCl into the polymer matrix. The compact morphology and absence of large voids confirm effective polymer-filler compatibility and the formation of a robust composite network, which is beneficial for ion transport and interfacial stability. Higher-magnification SEM images further confirm the homogeneous distribution of MPCl in the ETPTA matrix (Figure S2).

The thermal behavior of the GPE-Eh membrane was analyzed via TG–DSC (Figure 1e). A total mass loss of ~64.21 wt% is observed from 50 °C to 400 °C. The initial drop below 120 °C is attributed to the evaporation of residual liquid electrolyte, confirming the membrane’s electrolyte uptake and presence of volatiles. Between 120 and 340 °C, the weight remains stable, indicating good thermal stability of the ETPTA network and MPCl filler.

Above 340 °C, a further mass loss occurs, corresponding to the decomposition of the perovskite phase and polymer matrix.

XRD patterns of MPCl powder and all electrolyte membranes are presented in Figure 1f. The pristine MPCl shows sharp characteristic reflections consistent with its cubic perovskite crystal structure, indicating high crystallinity. In the GPE-Eh membrane, multiple strong diffraction peaks matching MPCl remain visible, confirming its preserved crystalline phase within the polymer matrix. Notably, several additional diffraction signals appear in the GPE-Eh pattern, which are not assignable to pure MPCl. These signals are likely due to secondary crystalline domains formed by local interactions between MPCl particles and the ETPTA–SA polymer matrix, or minor hydrated or degraded species produced during electrolyte soaking. Similar multi-phase features have been reported in perovskite–polymer hybrid systems [33,34], where confined crystallization or partial ion exchange can lead to new reflections. In contrast, GPE-El shows broad, weak reflections due to lower MPCl loading and partial amorphization, while GPE-P shows only a diffuse background due to its predominantly amorphous nature and the absence of MPCl. These results together confirm the successful incorporation of MPCl and the structural evolution among different compositions.

Figure 1g presents the FTIR spectra of the MPCl, GPE-P, GPE-El, and GPE-Eh membranes. The MPCl spectrum shows distinct bands near 900–600 cm^−1^ corresponding to Pb–Cl stretching vibrations. These features are partially retained in both GPE-El and GPE-Eh, suggesting the structural preservation of MPCl in the hybrid membranes. In GPE-El and GPE-Eh, additional absorption bands are observed near 1720 cm^−1^ and 1100–1000 cm^−1^, attributed to C=O stretching and C–O–C vibrations from the ETPTA network, respectively. The intensity of these peaks increases with higher MPCl content, indicating enhanced interaction between the polymer matrix and inorganic filler. Compared to GPE-P, which shows typical PEO-related peaks, the hybrid membranes exhibit more complex spectral features, confirming their distinct chemical composition and successful inorganic–organic integration.

To further evaluate the mechanical robustness of the composite electrolyte, tensile tests were conducted on the GPE-Eh membrane. As shown in Figure S3, the membrane demonstrates a tensile strength of 0.76 MPa and a breaking elongation of 9.2%, with an estimated Young’s modulus of approximately 8–9 MPa. These results confirm that the UV-crosslinked ETPTA matrix, though relatively soft, provides sufficient mechanical support for battery integration and operation.

**Figure 2:**
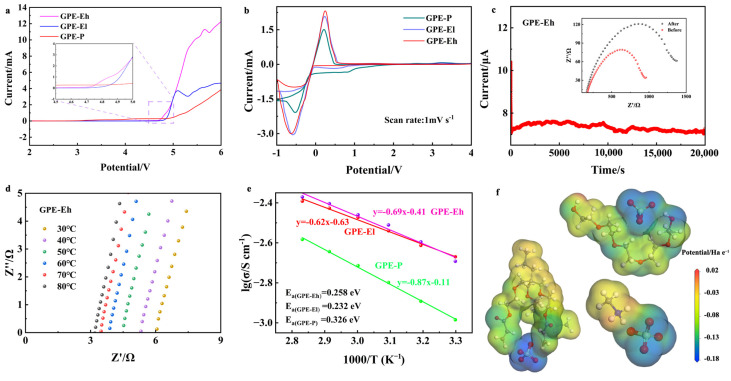
(**a**) LSV curves of GPE-P, GPE-El, and GPE-Eh; (**b**) CV curves of Na|GPE|SS half cells at 1 mV s^−1^; (**c**) DC polarization curve of GPE-Eh and Nyquist plots before/after polarization; (**d**) Nyquist plots of GPE-Eh at different temperatures; (**e**) Arrhenius plots and calculated activation energies of all samples; (**f**) electrostatic potential (ESP) maps of GPE components from DFT simulation.

The electrochemical performance of the composite electrolytes was systematically investigated and the results are summarized in Figure 2. As shown in Figure 2a, linear sweep voltammetry (LSV) was used to evaluate the electrochemical stability window of the electrolytes. The GPE-P membrane shows a gradual current increase beginning near ~4.7 V (vs. Na^+^/Na), indicating the onset of electrolyte oxidation. Although no sharp peak is observed, the deviation from baseline suggests limited oxidative stability. In contrast, the crosslinked ETPTA-based membranes exhibit significantly improved anodic stability, with GPE-El and GPE-Eh reaching higher decomposition voltages of ~4.8 V and ~4.9 V, respectively. This enhancement is attributed to the synergistic effect of the robust ETPTA network and the structural stabilization provided by the MPCl filler.

Figure 2b shows the cyclic voltammetry (CV) curves of Na | GPE | SS half cells at a scan rate of 1 mV s^−1^. All membranes exhibit distinct redox behavior and electrochemical responses within the voltage range of −1 to 4 V vs. Na^+^/Na. Notably, the GPE-Eh membrane displays a broader electrochemical window and more stable current response, suggesting enhanced anodic stability and interfacial compatibility. In contrast, GPE-P shows increased current fluctuations and the earlier onset of side reactions, indicating limited electrochemical stability under polarization. The stable and symmetrical CV shape of GPE-Eh implies lower interfacial resistance and better Na^+^ transport kinetics, benefiting from the perovskite–polymer hybrid network.

The ionic transport characteristics were further studied by DC polarization, as shown in Figure 2c. The GPE-Eh membrane reaches a steady-state current after polarization, and its sodium-ion transference number (*t_Na_*^+^) is calculated to be 0.66, which is markedly higher than GPE-El (0.58) and GPE-P (0.39). This result demonstrates that the incorporation of perovskite MPCl and the formation of a crosslinked polymer–inorganic framework enhance Na^+^ selectivity and suppress anion mobility. The inset Nyquist plots before and after polarization confirm good interfacial stability.

Figure 2d–f displays the Nyquist plots of the GPE-Eh membrane measured at various temperatures from 30 °C to 80 °C, showing a clear decrease in bulk resistance with increasing temperature. The corresponding Arrhenius plots are presented in Figure 2e, from which the ionic conductivity and activation energy were extracted. Among the samples, GPE-Eh exhibits the highest room-temperature ionic conductivity of 2.14 × 10^−3^ S·cm^−1^, significantly outperforming both the GPE-El (1.94 × 10^−3^ S·cm^−1^) and the conventional PEO-based GPE-P membrane (1.03 × 10^−3^ S·cm^−1^). This improvement is attributed to the presence of the perovskite MPCl phase in higher concentration, which enhances the formation of continuous and efficient ion-conductive channels throughout the crosslinked polymer network. In addition, the activation energy (*E_a_*) of GPE-Eh is calculated to be 0.258 eV, lower than that of GPE-P (0.326 eV) and GPE-El (0.281 eV), indicating more favorable Na^+^ transport kinetics in the hybrid electrolyte matrix. The reproducibility of the GPE-Eh membrane was confirmed across three independently prepared batches with similar electrochemical performance (Table S1).

To further elucidate the role of CH_3_NH_3_^+^ (methylammonium, MA^+^) in the composite structure, we analyzed the electrostatic potential (ESP) distribution obtained from DFT simulations (Figure 2f). In addition to the highly negative regions surrounding oxygen atoms in the polymer and halide anions (Cl^−^) in MPCl, the MA^+^ cation also presents polar functional groups—the amine (–NH_3_^+^) and methyl (–CH_3_)—which create local dipole fields. The –NH_3_^+^ group, carrying a partial positive charge, interacts electrostatically with surrounding electronegative atoms and may help stabilize Na^+^ ions near MPCl clusters through dynamic dipole–cation interactions. Moreover, the flexible orientation and dynamic nature of MA^+^ in the perovskite lattice may locally polarize the environment and reduce energy barriers for Na^+^ migration. This suggests that MA^+^ may not serve as a static conductor itself, but rather acts as a “dynamic facilitator” of ion hopping by altering the local potential landscape. Therefore, both the inorganic framework (PbCl_6_ octahedra) and organic components (MA^+^ and ETPTA) cooperatively contribute to building a percolated, hybrid Na^+^ migration network.

**Figure 3:**
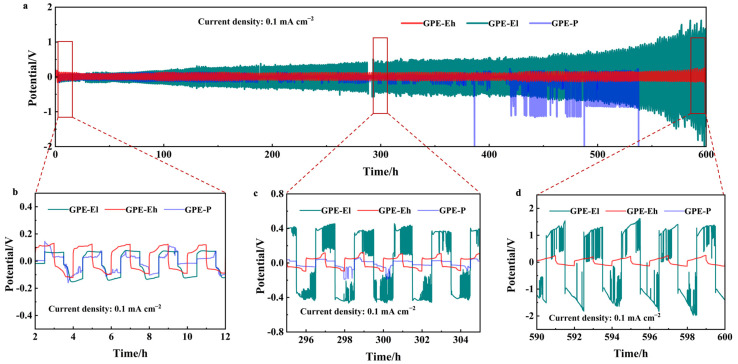
(**a**) Long-term sodium plating/stripping performance of Na|electrolyte|Na symmetric cells at 0.1 mA cm^−2^ over 600 h; (**b**–**d**) magnified views of voltage profiles in early (2–12 h), middle (296–304 h), and late stages (590–600 h) for GPE-P, GPE-El, and GPE-Eh membranes.

To assess the interfacial stability and long-term electrochemical durability of the composite electrolytes against sodium metal, symmetric Na|GPE|Na cells were assembled and tested under galvanostatic cycling at a current density of 0.1 mA cm^−2^. The cycling profiles over 600 h are shown in Figure 3a. Among the three membranes, the GPE-Eh cell exhibits the most stable and flat voltage profile throughout the entire test duration, maintaining polarization below 50 mV, without signs of short-circuit or voltage fluctuation.

In contrast, the GPE-P cell suffers from increasing overpotential and intermittent voltage spikes after ~400 h, eventually leading to unstable cycling due to dendrite growth or interfacial degradation. The GPE-El cell shows moderate stability but starts to fluctuate significantly beyond ~500 h, suggesting that the lower MPCl content provides insufficient structural reinforcement for the long-term suppression of sodium dendrite growth.

Magnified views of three typical intervals (early, middle, and late stage) are presented in Figure 3b–d. These show that GPE-Eh consistently maintains smooth, symmetrical sodium plating/stripping behavior, while GPE-El and GPE-P exhibit irregular voltage responses, with GPE-P displaying sudden noise and step-like changes, indicative of unstable interfacial contact.

These results confirm that the perovskite-rich, crosslinked polymer–inorganic matrix in GPE-Eh contributes to excellent interfacial compatibility with Na metal and effectively mitigates dendritic growth during prolonged cycling.

**Figure 4:**
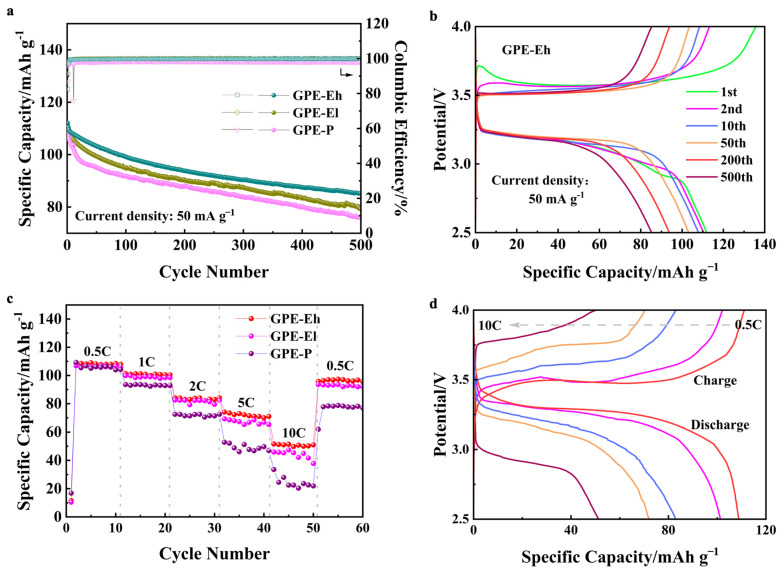
(**a**) Long-term cycling performance of Na|GPE|NVP cells at 50 mA g^−1^ for 500 cycles; (**b**) charge/discharge profiles of GPE-Eh at selected cycles; (**c**) rate performance of cells from 0.5 C to 10 C and return; (**d**) voltage profiles of GPE-Eh under different rates (0.5 C–10 C).

To evaluate the practical performance of the composite electrolytes in cell configuration, Na|GPE|NVP batteries were assembled and tested. As shown in Figure 4a, all cells exhibit gradually declining capacity over 500 cycles at 50 mA g^−1^, yet the cell using GPE-Eh maintains the most stable output, with a capacity retention of approximately 79%, clearly outperforming GPE-El and GPE-P, both of which retain <75% under identical conditions. Moreover, the Coulombic efficiency of the GPE-Eh cell remains above 98% throughout, indicating good reversibility. This suggests that the MPCl phase remains chemically and electrochemically stable under the cell operating conditions. The UV-crosslinked ETPTA matrix and dense polymeric network not only limit direct contact with moisture but also provide a confined, ion-buffered environment that likely mitigates decomposition under extreme voltage.

The voltage profiles of GPE-Eh during long-term cycling (Figure 4b) remain well-defined and symmetric, demonstrating minimal polarization and stable interfacial behavior. This suggests that the hybrid structure provides effective mechanical and ionic buffering under repeated cycling.

Figure 4c shows the rate performance of the cells. GPE-Eh again exhibits the best retention across all C-rates (from 0.5 C to 10 C), maintaining a higher percentage of its initial capacity at elevated rates and recovering over 89% of its capacity when returned to 0.5 C. In contrast, GPE-P suffers from a sharp capacity drop and incomplete recovery, reflecting limited ion transport and poor interfacial resilience.

The corresponding voltage curves (Figure 4d) further confirm that GPE-Eh enables smooth and moderate polarization even at high rates, indicative of stable ionic conductivity and robust electrode/electrolyte contact under dynamic conditions.

Furthermore, a full-cell configuration was assembled using an NVP cathode and a hard carbon anode to evaluate the practical performance of the GPE-Eh membrane. As shown in Figure S4a, the cell delivered an initial capacity of ~111.2 mAh g^−1^ at 50 mA g^−1^, with 86.3% capacity retention after 45 cycles. The stable charge/discharge voltage profiles (Figure S4b) further confirm the electrolyte’s compatibility and durability under practical operation.

A preliminary cost comparison of raw materials and processing steps is provided in the Supporting Information (Section S2), indicating the good scalability of this system.

## 4. Conclusions

In conclusion, the integration of perovskite MPCl into a UV-crosslinked ETPTA matrix enables the formation of an effective inorganic–organic composite electrolyte with enhanced ionic conductivity, wide electrochemical stability, and improved interfacial behavior. Among the designed systems, GPE-Eh demonstrated the most promising performance in both symmetric and asymmetric cells, benefiting from the synergistic combination of the highly conductive MPCl phase and the robust polymer framework. Specifically, GPE-Eh exhibited a high ionic conductivity of 2.14 × 10^−3^ S·cm^−1^ at room temperature, a low activation energy of 0.258 eV, and a sodium-ion transference number of 0.66. It also maintained over 83.8% of its capacity after extended cycling and showed excellent rate performance and reversibility. This work illustrates a feasible design strategy for next-generation quasi-solid-state sodium-ion batteries and offers valuable insights into the role of perovskite structures in polymer-based electrolytes.

## Data Availability

The original contributions presented in the study are included in the article and Supplementary Materials; further inquiries can be directed to the corresponding author.

## Supplementary Materials

The following supporting information can be downloaded at: https://www.mdpi.com/article/10.3390/ma18133106/s1, Figure S1: Photograph of the electrolyte membrane prepared with ETPTA:MPCl:SA = 4:21:1 after soaking in liquid electrolyte, showing significant cracking due to excessive filler content; Figure S2: High-magnification SEM image of the surface morphology of the GPE-Eh membrane, showing uniform MPCl dispersion within the ETPTA matrix; Table S1: Ionic conductivity and electrochemical stability of GPE-Eh membranes synthesized from three independent batches, demonstrating good reproducibility; Figure S3: Tensile stress–strain curve of the GPE-Eh membrane. The membrane exhibits a tensile strength of 0.76 MPa and an elongation at break of 9.2%, indicating moderate flexibility and acceptable mechanical integrity for practical handling in sodium-ion batteries; Figure S4: (a) Cycling performance and coulombic efficiency of a NVP|GPE-Eh|HC full cell at 50 mA g^−1^. (b) Galvanostatic charge/discharge profiles of the same NVP|GPE-Eh|HC full cell at the 2nd, 15th, and 45th cycles.
