# Supplementary material for: Composite Polymer Electrolytes with Tailored Ion-Conductive Networks for High-Performance Sodium-Ion Batteries
Source: Materials (Basel). 2025 Jul 1;18(13):3106. doi: 10.3390/ma18133106 (PMC12251254; doi:10.3390/ma18133106)
Supplement: Supplementary file 1 [file materials-18-03106-s001.zip › materials-3690679-supplementary.pdf]

Supporting information for

Composite Polymer Electrolytes with Tailored Ion-Conductive Networks for High-Performance Sodium-Ion Batteries

Section S1. Supporting Figures and Tables

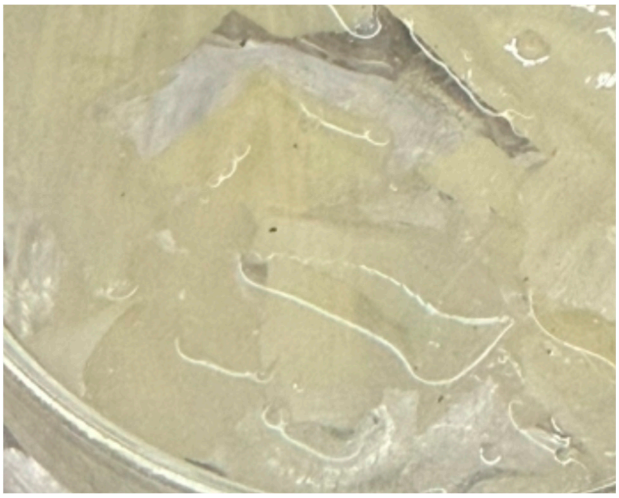

Figure S1. Photograph of the electrolyte membrane prepared with ETPTA:MPCl:SA = 4:21:1 after soaking in liquid electrolyte, showing significant cracking due to excessive filler content.

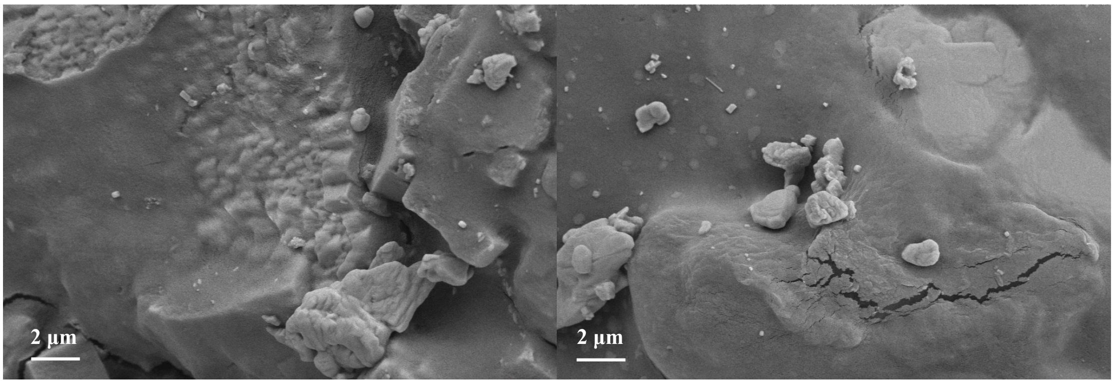

Figure S2. High-magnification SEM image of the surface morphology of the GPE-Eh membrane, showing uniform MPCl dispersion within the ETPTA matrix.

Table S1. Ionic conductivity and electrochemical stability of GPE-Eh membranes synthesized from three independent batches, demonstrating good reproducibility.

GPE-Eh Batches	Ionic Conductivity ($S \cdot cm^{-1}$)	Electrochemical Stability (V vs. Na^{+}/Na)
The first batch	2.11×10^{-3}	~4.9
The second batch	2.14×10^{-3}	~4.9
The third batch	2.18×10^{-3}	~4.8

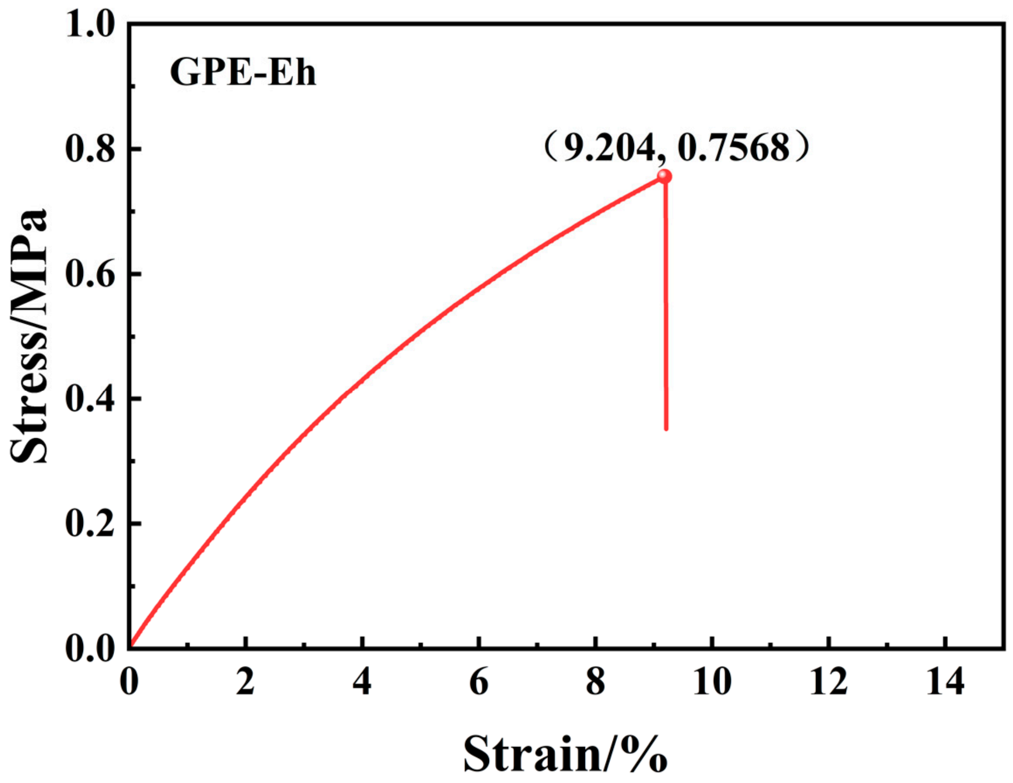

Figure S3. Tensile stress–strain curve of the GPE-Eh membrane. The membrane exhibits a tensile strength of 0.76 MPa and an elongation at break of 9.2%, indicating moderate flexibility and acceptable mechanical integrity for practical handling in sodium-ion batteries.

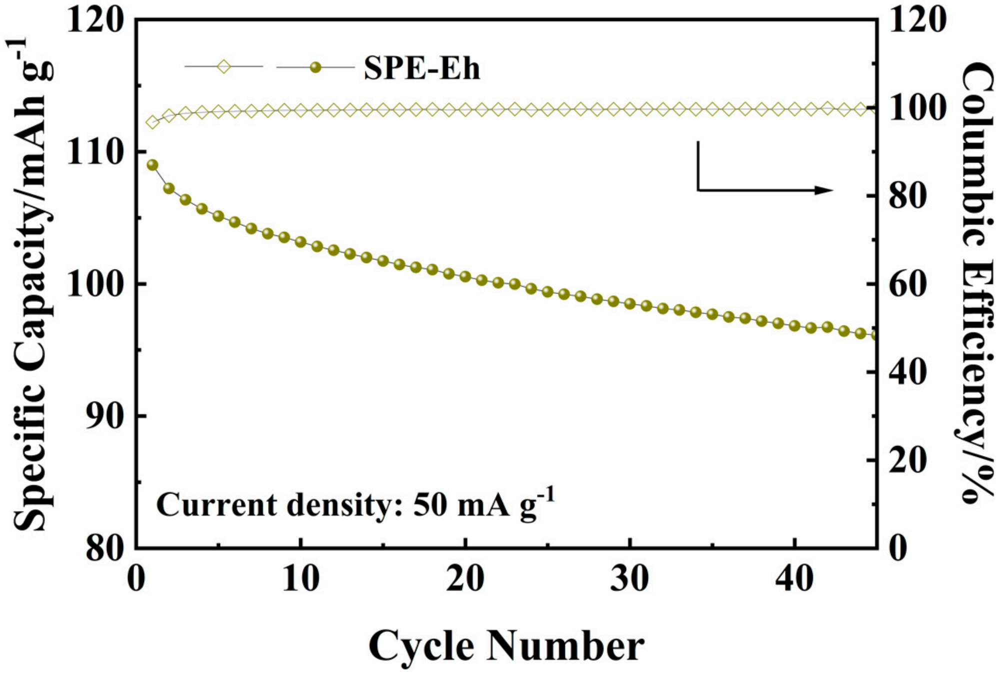

(**a**)

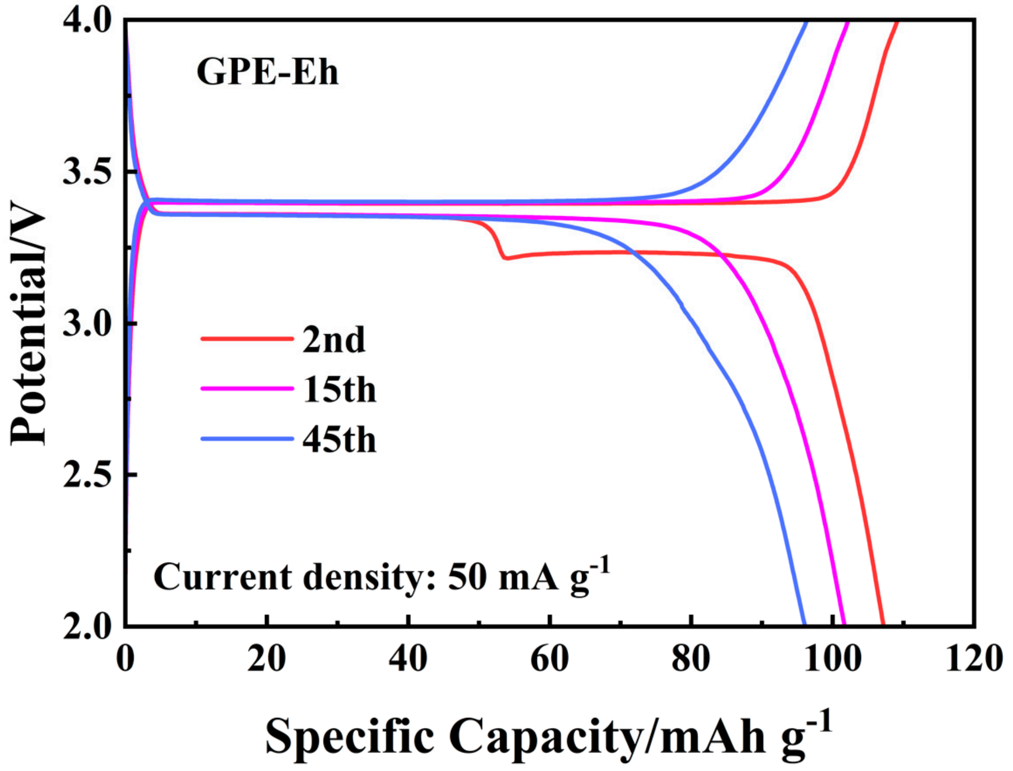

(**b**)

Figure S4. (**a**) Cycling performance and coulombic efficiency of a NVP|GPE-Eh|HC full cell at 50 mA g^{-1}. (**b**) Galvanostatic charge/discharge profiles of the same NVP|GPE-Eh|HC full cell at the 2nd, 15th, and 45th cycles.

Section S2. Cost Evaluation of the Composite Electrolyte Components

To assess the cost feasibility of the ETPTA–MPCl–SA composite gel-polymer electrolytes, we provide an estimation based on laboratory-scale reagent procurement:

Lead(II) chloride ($PbCl_2$): ¥71 per 100 g (~US $9.8/100 g, or ~US $98/kg)

Sodium alginate (SA): ¥57 per 25 g (~US $31/100 g, or ~US $310/kg)

Methylammonium chloride (MACl): ¥26 per 100 g (~US $3.6/100 g, or ~US $36/kg)

Although sodium alginate is relatively expensive per gram, it constitutes only a minor fraction of the composite formulation. The major inorganic filler, $PbCl_2$, is reasonably priced and used in moderate amounts. When considering the low weight per membrane and the UV-curing strategy adopted for in-situ film formation, the overall material and processing costs are expected to be substantially lower than those of conventional PEO-based solid electrolytes, which often require thermal annealing, moisture-sensitive handling, or inert gas protection.

This cost-efficient preparation route, combined with the promising electrochemical and mechanical performance, supports the practical scalability of our composite GPE system.
